# Identification of Ruminal Fermentation Curves of Some Legume Forages Using Particle Swarm Optimization

**DOI:** 10.3390/ani13081339

**Published:** 2023-04-13

**Authors:** Valiollah Palangi

**Affiliations:** Department of Animal Science, Faculty of Agriculture, Ege University, Bornova, Izmir 35100, Türkiye; valiollah.palangi@ege.edu.tr

**Keywords:** legume forages, fermentation curves, mathematical model

## Abstract

**Simple Summary:**

Forage plants are important for ruminant nutrition, so identifying their quality and nutritional value is effective in describing animal nutrition. A ruminal microbe attaches itself or is within close proximity to the surfaces of particulate substrates (primarily the inner surfaces) to digest them. However, incubation in the rumen can result in significant changes in the number of attached microbes. Nonlinear models may provide more accurate and comprehensive descriptions of feed fermentability. Improving the model suitability and validating the model are enhanced with low iterations. However, particle swarm optimization is the novelty of this study since it has never been used to study the digestive process with the above models.

**Abstract:**

The modeling process has a wide range of applications in animal nutrition. The purpose of this work is to determine whether particle swarm optimization (PSO) could be used to explain the fermentation curves of some legume forages. The model suited the fermentation data with minor statistical differences (R^2^ > 0.98). In addition, reducing the number of iterations enhanced this method’s benefits. Only Models I and II could successfully fit the fermentability data (R^2^ > 0.98) in the vetch and white clover fermentation curve because the negative parameters (calculated in Models III and IV) were not biologically acceptable. Model IV could only fit the alfalfa fermentation curve, which had higher R values and demonstrated the model’s dependability. In conclusion, it is advised to use PSO to match the fermentation curves. By examining the fermentation curves of feed materials, animal nutritionists can obtain a broader view of what ruminants require in terms of nutrition.

## 1. Introduction

Forages are obtained from forage plants and natural or artificial meadows [1]. In order to feed ruminant animals in an economical and physiologically sound manner, forage must be used. Nutritional demands are partially satisfied by forages that also provide some protection against some metabolic illnesses. Animal feed products with a high amount of nutritional forage are high-quality, and crucial to the state economy and the quality of life of its citizens [2,3]. In order to fit multiple mathematical models and choose the best-suited to represent the in vitro incubation of feeds, reliable estimates of fermentation parameters are necessary. Fermentation parameters are significant components of rumen models [4,5]. As fermentation parameters are positively correlated with digestibility, energy content, and possible reductions in the fill effect in forage, it may be possible to classify DMI intake between species by presenting each forage as a single forage or presenting each forage as a preferred food in a cafeteria test [6].

Different nutritional sciences utilize modeling to explain various phenomena and express connections among the components that affect a biological process [7]. There are numerous methods for estimating feed-quality proportions; however, the detailed studies of biological processes could offer valuable insight into the dynamics of biological processes and reveal their other dimensions [8,9]. Nonlinear models could be used to obtain more detailed and comprehensive information on the degradability of feeds. Data from an in vitro technique showed that many mathematical models have been created as prospective candidates to characterize the potential ruminal fermentability of feeds. Various models have been applied to estimate nutrient degradability parameters, and hypotheses have been developed concerning the biological processes that drive the separation of ruminal digestion in order to develop mathematical models capable of predicting rumen disappearance [10,11]. Different artificial-intelligence techniques have been extensively applied to a variety of sciences [12,13]. One of these techniques, the particle swarm optimization (PSO) methodology, is most applicable to the study of fermentation curves. Particle swarm optimization (PSO) adjusts each particle’s position according to its previous position, and the locations in its immediate vicinity on the basis of previous experiences. The first iteration of the algorithm was motivated by a simulation of a flock of birds engaged in social behavior while searching for food. Each bird adjusts its location on the basis of its prior location or by coming into contact with the bird that located the best food. Each bird in the method is a potential solution in the issue search space, which includes elements of position, velocity, communication with other birds, and the memory of prior positions. Each bird can make decisions using the information [14]. With the PSO method, fermentation curves are better fitted, and the method can reach convergence and reduce errors with intelligent parameter estimation.

However, feed evaluation might benefit more from defining digestible models and showing their degradation curves. Animal nutritional science can be viewed from a fresh perspective by integrating recent developments in swarm intelligence with ruminal degradation and applying these sciences to improving the curves, which can reduce common errors in this area. This study aims to identify ruminal fermentation curves by optimizing the particle swarm. The digestion rate depends on how long the food remains in the digestive tract. As a result, changes in the fermentation process can be depicted by segmenting feed into the following parts: (1) quickly fermentable fraction, (2) slowly fermentable fraction, and (3) indigestible. We hypothesized that nonlinear models could be used to obtain more detailed and comprehensive information regarding the fermentability of feeds. The objective of this study was to use particle swarm optimization to produce the fermentation curves of hay from alfalfa (*Medicago polymorpha*), vetch (*Vicia villosa*), and white clover (*Trifolium repens*).

## 2. Materials and Methods

### 2.1. Data Collection

Using the raw data of Palangi and Macit [15], the proposed approach was validated. In this calculation, alfalfa (*Medicago polymorphia*), clover (*Trifolium repens*), and vetch (*Vicia villosa*) were used for gas production in vitro as in [15]. The volume of the gas was measured and sampled after 3, 6, 12, 24, 48, 72, and 96 h of incubation. As mentioned in the previous article, the control treatments’ raw data were used for this experiment. The degradability statistics was organized according to various incubation times, and incorporated in the software’s editor window.

### 2.2. Particle Swarm Optimization (PSO)

Collective intelligence optimization algorithms, such as PSO, are one type of intelligence algorithm. An initial population is predicated on this type of dynamic computing and other dynamic approaches. The process begins with a particle or component that consists of a population of randomly generated solutions. The fundamental concept can be found in a 1987 paper by Reynolds that simulated the movement of birds in flocks [16]. According to that article, the collective behavior of the birds was driven by three simple movements: alignment, separation, and cohesion. Due to the strong performance of this algorithm, it attracted much attention. Consequently, indepth research was performed, and several varieties of the method presented [17,18,19]. Due to its generality, this technique was applied in many other domains [20,21,22].

**Figure 1:**
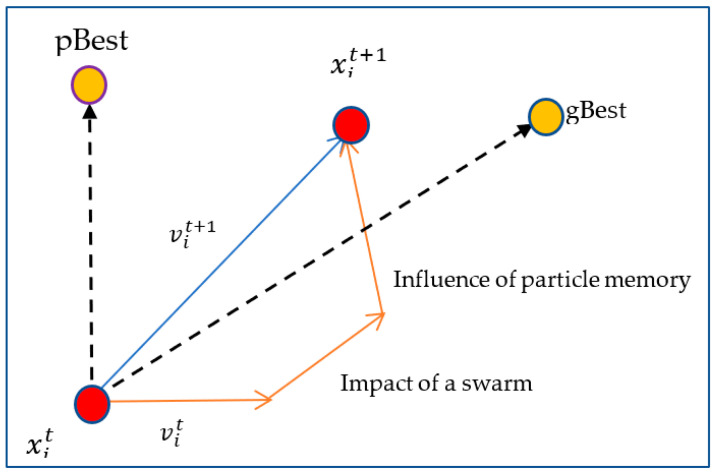
An individual particle’s movement in a particular step.

On the basis of a simulation of birds searching for food in a flock, the first version of the algorithm was developed. Additionally, each bird could use the information to make decisions. At every step, the PSO algorithm moves particles toward a better position through a finite number of steps. In each step, each particle moves according to one of three tendencies: to continue its current motion, to move to the best position it had previously found (pBest), or to move to the best position that the whole system had found (gBest). Figure 1 shows how each particle moves in a specific direction.

### 2.3. Curve Fitting

From the collected data using the in vitro approach, various mathematical models were created as prospective candidates to characterize the kinetics of feed fermentation. The behavior of the model was examined using the first-order kinetics model (without and with a lag phase) [23]. The mathematical definition is as follows:

Model (I)—first-order kinetic model without a lag phase:

P = a + b (1 − e^−ct^) (1)

Model (II)—first-order kinetics model with lag phase:

P = a + b (1 − e^−c(t+L)^) (2)

It develops ideas about biological principles that characterize the percentage of rumen disappearance while using various models to predict nutrient degradability characteristics. These models can be used to estimate the ruminal digestion parameters of feed, which can subsequently be used to compare feeds or various nutritional systems [7].

Model (III)—Gompertz model:

P = a + b (K − K^exp(−ct)^/K − 1) (3)

Model (IV)—generalised Mitscherlich model:

P = a + b (1 − e^−c(t−L)−d(√t−√L)^) (4)

The degradability process can also be simulated using sigmoid models instead of linear models. In addition to linear models, nonlinear models may provide more accurate and comprehensive information about feed degradation. In order to calculate the initial residual sum of squares for all permutations of starting values, and to begin the iterations with the best set, the model uses many probable starting values on a grid by providing PSO with more than one starting values. The beginning points and ranges needed for the models were defined, and the models were identified using the editor’s tool script. For calculating the error values of the fitted curve, the goodness-of-fit function was used.

### 2.4. Optimal Problem-Solving Model

This part outlines the examination of the fitting issue, and a PSO-based approach to equation optimization fitting is described. The PSO algorithm must be updated to take into account the circumstances of the issue. The selection of the fitness function is the most crucial aspect for this algorithm to take into account. The algorithm’s main goal is to reduce the fitness function. Additionally, effective variables should be identified, and function minimization should be optimally modified. The PSO algorithm for fitting the curve is as follows.

### 2.5. Proposed Algorithm

The algorithm’s main goal is to identify the relation that could best represent the necessary data. The quantification of random particles is the algorithm’s first step. Values a, b, c, L, d, and k in each particle can be combined to form the ideal solution. In the PSO algorithm, one of the parameters is population size, which must be changed. When the population is small, there is a lower chance of finding the best answer, and when the population increases, the algorithm takes longer to execute. As a result, a reasonable number ought to be taken into account. Using the values of variables for each particle, the fitness function value is calculated. The results represent the best particle value, pBest. The minimal value of the best general particle was also be saved as gBest.

Cost is another component of the PSO algorithm. To determine the cost, the problem’s circumstances must be taken into account. Finding a relation that could fit all of the problem data is the aim. Hence, any departure from the function diagram can be seen as a mistake. The overall number of errors may be viewed as a cost, depending on the specifics of the issue. An overall reduction in errors is the goal of cost optimization implementation.

## 3. Results and Discussion

The online version of MATLAB was employed in this study. The coefficients of the equations were divided into a four-dimensional search space for the efficient operation of the PSO algorithm’s efficient operation. Table 1 was used to set the PSO algorithm’s parameters. As illustrated in Figure 1, the iteration of in vitro gas production data showed that legume forages had the lowest iteration. As opposed to linear citizenship equations, which can be solved in one step, nonlinear citizenship equations can only be solved after successive iterations. Iterations such as these are due to the software assuming different parameter values at the beginning, and then fitting the data with a curve on the basis of these values. To achieve the best fit between the equation and the data, this method uses a trial-and-error procedure [24,25]. By applying this method, we could more quickly find equations that are suitable for describing the behavior of models [26]. A model’s behavior is most accurately described by the method used in the following figures. Model evaluations are relative processes that include all readily available criticism methods and involve the evaluator from their perspective.

**Figure 2:**
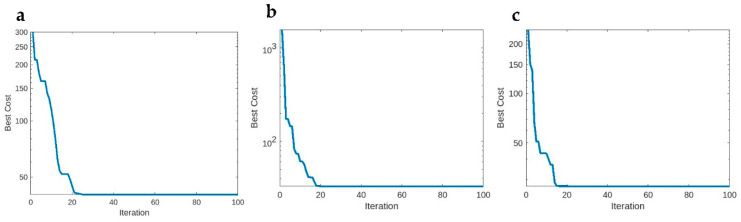
Best cost of in vitro gas production for legume forages (**a**) alfalfa, (**b**) vetch, and (**c**) clover.

**Figure 3:**
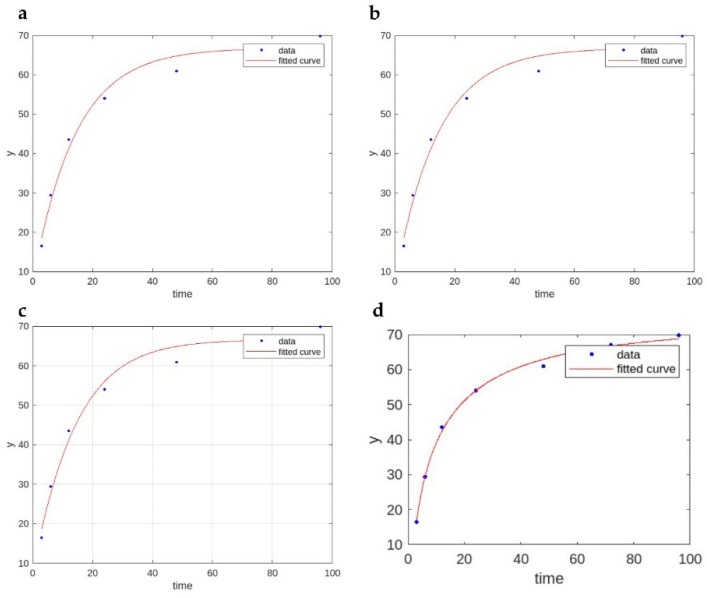
Ruminal fermentation curve for alfalfa forages. (**a**) Model I, (**b**) Model II, (**c**) Model III, and (**d**) Model IV; X = incubation time, Y = fermentation amount.

**Figure 4:**
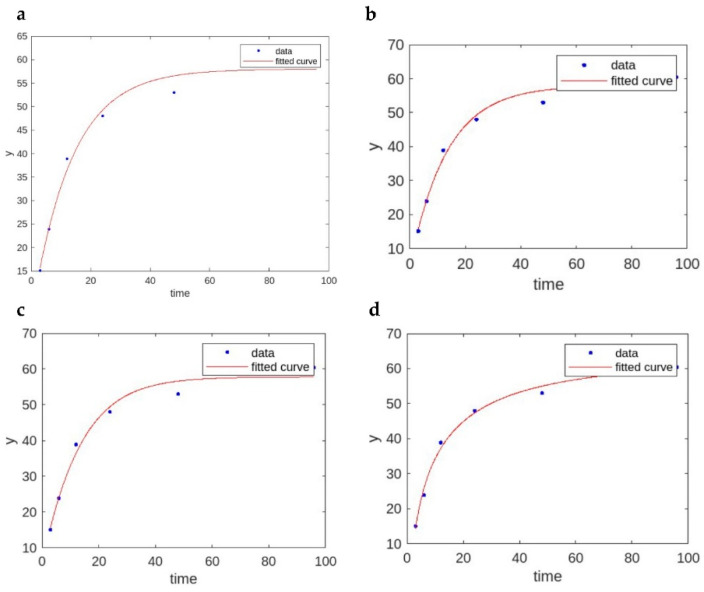
Ruminal fermentation curve for vetch forages. (**a**) Model I, (**b**) Model II, (**c**) Model III, and (**d**) Model IV; X = incubation time, Y = fermentation amount.

**Figure 5:**
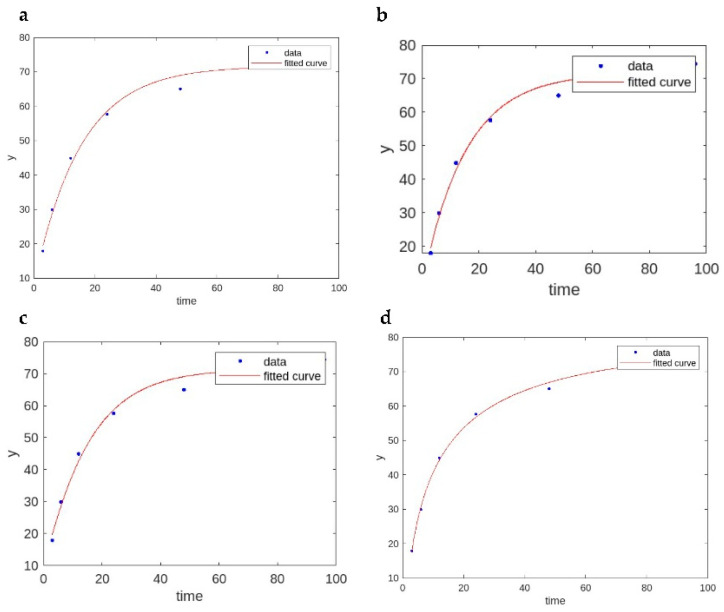
Ruminal fermentation curve for clover forages. (**a**) Model I, (**b**) Model II, (**c**) Model III, and (**d**) Model IV; X = incubation time, Y = fermentation amount.

As illustrated in Figure 2, the best cost of in vitro gas production for legume forages was obtained with the least number of iterations. As opposed to linear equations of subordination that can be solved in one step, nonlinear equations of subordination can only be solved with successive iterations that are the result of the software assuming different initial values for the parameters. Then, they are fitted to the curve on the basis of these values, and often on the basis of experience and previous testing results. For each parameter, an initial value must be considered before solving the subparameter. As long as the new values for the parameters do not result in a better fit (residual value with less error) than that of the previously selected values, the process is repeated. By providing more parameters to the evaluator, the PSO method reduces the evaluator’s opinion. The number of required iterations to reach the solution is also considered in examining the model’s behavior, so a higher number indicates a less competent model in fitting the data. Reducing the number of iterations increases the validity of the model and shows its suitability. Figure 3, Figure 4 and Figure 5 show the results of the ruminal fermentation curve for alfalfa, vetch, and clover.

There are several control parameters that affect the fundamental PSO, such as the problem size, the number of particles, the acceleration coefficients, the inertial weights, the neighborhood size, and the number of iterations. Some of these parameters are random values that scale the contribution of cognitive and social components. In Table 2, some of them are listed.

Table 2 lists the predicted parameters for a mathematical model fitted with PSO that describes in vitro gas production data for legume forages. During model evaluation, all available methods are used to review the model, but this is a relative process becaue the evaluator is involved. In general, none of the proposed models is complete; they are usually incomplete in some aspects. In addition to examining the behavior and statistical tests of models to choose the best, the biological significance of the estimated metaparameters and their justification were also considered.

As a result of the high concentrations of slowly fermentable fraction b (especially in clover), passage contents were increased. Forages should have less of Part b, but for feeds containing high-value biological proteins, Part b should be increased, so that most of the protein could pass through rumen digestion and be absorbed. Gas production during in vitro incubation indicates ruminal degradation and microbial activity [27], and a higher b fraction indicates higher rumen undegradable protein (RUP) content [28]. Yuan and Wan [29], and Ayaşan et al. [30] reported negative values for Part a, which is consistent with the findings of the current investigation. However, negative values for Parts a and b are not biologically acceptable. The lag time in the study of Wang et al. [31] varied between −0.14 and 0.75 (h), which is consistent with our research. The calculated fermentation rate constant (c) in the study of Eseceli et al. [32] was parallel with that in our study. According to Esen et al. [33], who studied the nutritive value of *Styrax officinalis* L. shrubs at four stages of maturity, namely, preflowering (PF), flowering (FL), seed linkage (SL), and fruiting (FR), reported “a” fractions of 31.50, 21.13, 11.60 and 22.70, respectively, which were higher than those in our results. Another study [34] showed that alfalfa hay had 56.1% of the slowly fermentable fraction, which is consistent with our findings. These achievements are in agreement with the reports of Palangi et al. [23], who noted that the fermentation curves for legume forages vary according to their maturity and chemical composition. As previously discussed by Bayatkouhsar et al. [35], cutting alfalfa in the afternoon as opposed to in the morning may result in higher concentrations of soluble carbohydrates, leaf content, and true protein, which may impact the fermentation pattern using the gas production method. Therefore, harvest time can be a source of changes in fermentation parameters.

Comparing the various models for estimating the fermentation characteristics of legume forages shows that Models I and II, described by Orskov and McDonald [36], attained convergence, whereas others were not biologically acceptable due to the predicted negative values. Among the models that had attained convergence, Model I, without lag time, had the greatest fit even though its performance was marginally superior to that of the other models. Despite only being able to fit alfalfa, our results reveal that Model IV was the most successful of all fitted models due to its higher adjusted R squared (Adj R^2^) value. Model IV could only fit the alfalfa fermentation curve, which had higher R values and demonstrated the model’s dependability. A longer delay time (5.73) indicated that the inclusion of alfalfa in the combinations may have delayed the microbial fermentation of digestible components or may have caused a change in the site of digestion. The slower in vitro fermentation of the vetch fractions was likely caused by the increase in acid detergent lignin (ADL) concentration, which constrained access to the cell content, and decreased the degradation of nutrients.

The mean square of the prediction error was calculated as the sum of the squares of the difference between the late predicted and observed values divided by the number of observations, and its smaller numerical value indicates the suitability of the model. Models I and II had the least sum of squares due to error (SSE) for vetch forages, so these models had high merit in fitting this forage. In connection with the components of the mean square of the prediction error, the error in terms of the central tendency shows how much the average of the predicted values deviated from the observed values. The error in terms of subordination measures the deviation of the least-squares subordination coefficient from the first (the number that is obtained if the predicted values are completely accurate); when it is large, it shows the inability of the model to predict the dependent variable. The error in terms of dispersion is the expression of the dispersion in the observed values that remains after removing errors in terms of subordination and the central tendency.

Glycocellulose feeds undergo ruminal fermentation in a dynamic system that is influenced by interactions among animals, their diet, and the microbial population. The ability of an animal to digest raw fibers in the rumen often depends on the animal’s digestive capacity in relation to the activity and digestibility of the rumen, and the amount of consumed forage. This has led to the development of different mathematical models describing the ratio of rumen disappearance as a result of applying different models to estimate feed degradability parameters and to formulate hypotheses related to the biological principles that govern the separation of ruminal digestion. In this way, different feed materials or nutritional systems can be compared by estimating ruminal digestion parameters. By studying the digestive models of feed materials, animal nutritionists could more accurately determine the needs of ruminant animals. The performance of each model in terms of the fitting fermentation curves was around average, and the statistical evaluations of the models showed only slight variations. However, depending on the model’s structure and the used parameters, the estimated parameters varied among the models.

## 4. Conclusions

The study findings clearly show that the R^2^ and adjusted R^2^ values were appropriately high. R^2^ indicates how well a curve or line fits the data points. In addition to indicating how well terms fit a curve or line, the adjusted R^2^ adjusts for the number of terms in a model. Therefore, these parameters show that the curves were well-fitted. Fewer required consecutive iterations to solve the model quickly also suggest that the algorithm quickly found the solution. As a result of the fewer iterations, this method was more efficient at solving the model. Therefore, fitting the fermentability curves with PSO is recommended, and the approach is easily adaptable to various models. It is sufficient to alter the fitness function and some parameter values. Therefore, this method could intelligently estimate values and optimize curve fitting with the initial parameters. Equations require that, after modifying the fitting equation in accordance with its formula, the remaining PSO algorithm parameters be tuned while maintaining the dimensions of the problem space. However, for Equation (4) (the generalized Mitscherlich model), we also need to enlarge the problem space to 6, which is equivalent to parameters a, b, c, L, d, and k. Additionally, the parameters of the algorithm were fine-tuned, and the fitness function was modified; this method was effective in achieving this goal due to its intelligence. To implement the calculated parameters for use in practice, the biological properties of the tested models should be taken into account given their similar performance; hence, the best models are recommended by matching the results with biological facts. As a result, the application of the method described in this work to other models is simple. There are different digestion models to investigate the process of the ruminal degradability of edible DM and CP. Different mathematical models allow for better parameter comparisons to understand the ideal biological relationships between feeds and feeding systems. In order to obtain more information for the right decisions in ruminant feed rations, it is recommended to fit other statistical models that estimate more parameters.

## Figures and Tables

**Table 1:** Particle swarm optimization parameters.

Parameter	Value	Description
nPop	500	Numerical value of particles
MaxIt	100	Maximal iteration count
*n*	7	*n* is the problem’s dimension
C_1_	1.5	PSO parameter C_1_
C_2_	2	PSO parameter C_2_
wdamp	1	Inertial weight damping ratio

**Table 2:** In vitro gas production parameters of legume forages using a mathematical model fitted with PSO.

	Parameters ^1^						SSE ^2^	R^2^	Adjusted R^2^	RMSE
	*a*	*b*	*c*	*L*	*d*	*k*				
Alfalfa										
Model I ^3^	7.171	59.61	0.07045	-	-	-	40.2406	0.9832	0.9748	3.1718
Model II	23.36	43.42	0.07046	0.3169	-	-	40.2406	0.9832	0.9664	3.6625
Model III	−211.6	58.47	0.07602	-	-	0.7505	44.6032	0.9814	0.9627	3.8559
Model IV	28.66	47.71	0.01097	5.73	0.3819	-	6.5756	0.9973	0.9918	1.8132
Vetch										
Model I	5.017	52.97	0.07508	-	-	-	27.2274	0.9851	0.9777	2.6090
Model II	18.94	39.05	0.07509	0.305	-	-	27.2274	0.9851	0.9702	3.0126
Model III	−174.1	51.92	0.08169	-	-	0.7322	29.8218	0.9837	0.9674	3.1529
Model IV	23.46	41.78	−0.0105	5.2	0.3991	-	11.8790	0.9935	0.9805	2.4371
Clover										
Model I	8.191	63.5	0.06554	-	-	-	33.0409	0.9881	0.9822	2.8741
Model II	25.78	45.91	0.06555	0.3244	-	-	33.0409	0.9881	0.9763	3.3187
Model III	−234.7	62.38	0.07069	-	-	0.759	37.0349	0.9867	0.9734	3.5135
Model IV	32.17	50.77	−0.009137	6.464	0.3488	-	5.868	0.9979	0.9937	1.7129

^1^ *a* = rapidly soluble fraction (%); *b* = slowly fermentable fraction (%); *c* = fermentation rate constant (%/h) of fraction ‘b’; *L* = lag time (h); *d* = is the parameter pertaining to the variable fractional rate of fermentation; *k* = slope, or fermentation rate coefficient (h^−1^); ^2^ SSE = sum of squares due to error; R-squared = the proportion of the variation in the dependent variable that is predictable from the independent variable; Adj R-squared = a modified version of R-squared that was adjusted for the number of predictors in the mode; RMSE = root-mean-square error. ^3^ Model I, first-order kinetic model without lag phase; Model II, first-order kinetic model with lag phase; Model III, Gompertz model; Model IV, generalized Mitscherlich model.

## Data Availability

Not applicable.

## References

[1] Aliu S., Rusinovci I., Fetahu S., Zeka D. (2019). Performance of Forage Crops and Grass Mixtures in Kosovo. Grasses and Grassland Aspects.

[2] Mahanta S.K., Garcia S.C., Islam M.R. (2020). Forage based feeding systems of dairy animals: Issues, limitations and strategies. Range Manag. Agrofor..

[3] Zhang X., Wang H., Guo X. (2021). Effects of total mixed ration with various silage on growth performance, serum parameters, ruminal fermentation, and bacteria community profile in beef cattle. Food Sci. Nutr..

[4] He Y., Sun X., You P. (2021). Animal, feed and rumen fermentation attributes associated with methane emissions from sheep fed brassica crops. J. Anim. Physiol. Anim. Nutr..

[5] Dong J.N., Li S.Z., Chen X., Qin G.X., Wang T., Sun Z., Zhen Y.G. (2021). Effects of different combinations of sugar and starch concentrations on ruminal fermentation and bacterial-community composition in vitro. Front. Nutr..

[6] Lagrange S., Lobón S., Villalba J.J. (2019). Gas production kinetics and in vitro degradability of tannin-containing legumes, alfalfa and their mixtures. Anim. Feed Sci. Technol..

[7] Palangi V., Besharati M. (2020). Validation of in situ disappearance curves utilizing mathematical models for incubating fish meal and cottonseed meal. Semina: Cien. Agrar..

[8] Do D.T., Le T.Q.T., Le N.Q.K. (2021). Using deep neural networks and biological subwords to detect protein S-sulfenylation sites. Brief. Bioinform..

[9] Palangi V. (2021). In situ ruminal degradation of sallow tree leaves using different mathematical models. Rev. MVZ Córdoba.

[10] Bannink A., Kar S., Schokker D., Dijkstra J. (2020). A conceptual approach to the mathematical modelling of microbial functionality in the rumen. Improving Rumen Function.

[11] McFadden L.J., Menendez H.M., Olson K., Brennan J.R., Ehlert K., Blair A. (2022). 4 Developing a Dry Matter Intake Prediction Equation for Grazing Animals based on Real-Time Enteric Emissions Measurements. J. Anim. Sci..

[12] Xu Y., Liu X., Cao X., Huang C., Liu E., Qian S., Zhang J. (2021). Artificial intelligence: A powerful paradigm for scientific research. Innovation.

[13] Marini F., Walczak B. (2015). Particle swarm optimization (PSO). A tutorial. Chemom. Intell. Lab. Syst..

[14] Bensingh R.J., Machavaram R., Boopathy S.R., Jebaraj C. (2019). Injection molding process optimization of a bi-aspheric lens using hybrid artificial neural networks (ANNs) and particle swarm optimization (PSO). Measurement.

[15] Palangi V., Macit M. (2021). Indictable mitigation of methane emission using some organic acids as additives towards a cleaner ecosystem. Waste Biomass Valorization.

[16] Reynolds C.W. (1987). Flocks, Herds and Schools: A Distributed Behavioral Model. ACM SIGGRAPH Comput. Graph..

[17] Coello C.C., Lechuga M.S. MOPSO: A Proposal for Multiple Objective Particle Swarm Optimization. Proceedings of the 2002 Congress on Evolutionary Computation, CEC’02 (Cat. No. 02TH8600).

[18] Ma Y.F., Zhang H.J. Contrast-Based Image Attention Analysis by using Fuzzy Growing. Proceedings of the Eleventh ACM International Conference on Multimedia.

[19] Cavalca D.L., Spavieri G., Fernandes R.A. (2018). Comparative Analysis between Particle Swarm Optimization Algorithms Applied to Price-Based Demand Response. Proceedings of the Artificial Intelligence and Soft Computing: 17th International Conference, ICAISC 2018.

[20] Chaubey N., Goree J., Lanham S.J., Kushner M.J. (2021). Positive charging of grains in an afterglow plasma is enhanced by ions drifting in an electric field. Phys. Plasmas.

[21] Nasrollahzadeh S., Maadani M., Pourmina M.A. (2021). Optimal motion sensor placement in smart homes and intelligent environments using a hybrid WOA-PSO algorithm. J. Reliab. Intell. Environ..

[22] Song S., Xiong X., Wu X., Xue Z. (2021). Modeling the SOFC by BP neural network algorithm. Int. J. Hydrog. Energy.

[23] Palangi V., Macit M., Bayat A.R. (2020). Mathematical models describing disappearance of Lucerne hay in the rumen using the nylon bag technique. South Afr. J. Anim. Sci..

[24] Sebaaly H., Varma S., Maina J.W. (2018). Optimizing asphalt mix design process using artificial neural network and genetic algorithm. Constr. Build. Mater..

[25] Woodhouse E.J., Collingridge D. (2019). Incrementalism, intelligent trial-and-error, and the future of political decision theory. An Heretical Heir of the Enlightenment.

[26] Lookman T., Balachandran P.V., Xue D., Yuan R. (2019). Active learning in materials science with emphasis on adaptive sampling using uncertainties for targeted design. NPJ Comput. Mater..

[27] Boussaada A., Arhab R., Calabrò S., Grazioli R., Ferrara M., Musco N., Cutrignelli M.I. (2018). Effect of Eucalyptus globulus leaves extracts on in vitro rumen fermentation, methanogenesis, degradability and protozoa population. Ann. Anim. Sci..

[28] Bachmann M., Kuhnitzsch C., Michel S., Thierbach A., Bochnia M., Greef J.M., Zeyner A. (2020). Effect of toasting grain silages from field peas (*Pisum sativum*) and field beans (*Vicia faba*) on in vitro gas production, methane production, and post-ruminal crude protein content. Anim. Nutr..

[29] Yuan J., Wan X. (2019). Multiple-factor associative effects of peanut shell combined with alfalfa and concentrate determined by in vitro gas production method. Czech J. Anim. Sci..

[30] Ayaşan T., Esen S., Cabi E., Eseceli H., Esen V.K. (2020). Effect of arbuscular mycorrhizal inoculation on the quality and in vitro gas production of einkorn wheat straw. South Afr. J. Anim. Sci..

[31] Wang M., Zhang F., Zhang X., Yun Y., Wang L., Yu Z. (2021). Nutritional quality and in vitro rumen fermentation characteristics of silage prepared with lucerne, sweet maize stalk, and their mixtures. Agriculture.

[32] Eseceli H., Ayasan T., Koç F., Esen V.K., Esen S. (2020). Nutrient and mineral contents, and in vitro digestibility of kermes oak (*Quercus coccifera* L.) and mock privet (*Phillyrea latifolia* L.). Alinteri J. Agric. Sci..

[33] Esen S., Koc F., Özdüven L., Eseceli H., Evren C., Karadağ H. (2022). In situ and in vitro nutritive value assessment of styrax officinalis l. as an alternative forage source for goat feeding. J. Agric. Sci..

[34] Mohammadabadi T., Chaji M., Direkvandi E., Alqaisi O. (2021). Effect of replacing alfalfa hay with Leucaena leucocephala (L. *Leucocephala*) leaves on in vitro gas production, digestibility and in situ degradability in buffalo. Acta Scientiarum. Anim. Sci..

[35] Bayatkouhsar J., Rezaii F., Ghanbari F., Rahchamani R. (2022). Morning vs. afternoon harvest time of alfalfa, clover, and barley affect the chemical composition and nutritional value of silage. Iran. J. Appl. Anim. Sci..

[36] Ørskov E.R., McDonald I. (1979). The estimation of protein degradability in the rumen from incubation measurements weighted according to rate of passage. J. Agric. Sci..

